# The Effect of a Biofeedback-Based Integrated Intervention for Older Adults with Orthostatic Hypotension: A Secondary Analysis on Psychological Health Outcomes in a Non-Randomized Pilot Trial

**DOI:** 10.3390/healthcare12212143

**Published:** 2024-10-28

**Authors:** Nahyun Kim, Jeonghwa Han, Hyunwook Kang

**Affiliations:** 1 College of Nursing, Keimyung University, Daegu 42601, Republic of Korea; drkim@kmu.ac.kr; 2 Department of Nursing, Kyungwoon University, Gumi 39160, Republic of Korea; jeonghwahan0@gmail.com; 3 College of Nursing, Kangwon National University, Chuncheon 24341, Republic of Korea

**Keywords:** aged, orthostatic hypotension, biofeedback, quality of life, depression, anxiety, fall

## Abstract

Background/Objectives: Aging-related physical changes and dysfunctions in the autonomic nervous system (ANS) often lead to orthostatic hypotension (OH) in older adults. OH negatively impacts both the physical and psychological well-being of those affected. Previous studies have demonstrated that the biofeedback-based integrated program (BBIP), a multicomponent intervention focused on heart rate variability biofeedback, effectively improves OH, as well as symptoms related to ANS function. This substudy aims to examine the effects of the BBIP on psychological health outcomes among community-dwelling older adults with OH. Methods: This study employed a non-randomized controlled trial design with a convenience sampling strategy. A total of 51 older adults with OH were recruited from two senior welfare centers and randomly assigned to either the intervention group (n = 27) or the control group (n = 24). The intervention group participated in a 12-week BBIP, which included weekly biofeedback sessions and group education on lifestyle modification to alleviate OH. Telephone counseling was also provided to promote compliance. Results: The intervention group showed significant improvements in health-related quality of life, depression, anxiety, and fall efficacy after the 12-week BBIP, whereas the control group exhibited no significant changes. There was a significant reduction in the percentage of participants in the intervention group reporting problems in all five dimensions of the EQ-5D (mobility, self-care, usual activities, pain/discomfort, and anxiety/depression). Conclusions: The BBIP was effective in improving the psychological health outcomes of older adults with OH. Future studies should explore the long-term effects of the BBIP using a larger sample size and a randomized controlled trial design.

## 1. Introduction

Orthostatic hypotension (OH) is a condition prevalent in older adults, predominantly due to physiological changes associated with aging, such as autonomic nervous system (ANS) dysfunction and arterial stiffness [1,2]. This problem is compounded by comorbid conditions and medications, including antidepressants and antihypertensives, which also contribute to OH [1]. According to a meta-analysis of epidemiological studies, OH affects 22.2% of community-dwelling older adults (pooled N = 24,997) [3]. Despite this high prevalence, only 2% of these individuals exhibit symptoms, leading to under-recognition and under-treatment [3].

Additionally, OH can lead to numerous physical and psychological health issues. Physical manifestations include dizziness, lightheadedness, cognitive impairment, and syncope—all results of reduced blood flow to the brain—and blurry vision due to reduced perfusion to the retina and visual pathways [1]. These symptoms increase the risk of falls, which in turn can lead to decreased daily activity due to fear of falling and resultant disability [4]. The nonspecific occurrence of dizziness is often linked with anxiety or panic [5].

Moreover, studies have demonstrated the association between mental health problems including depression or anxiety and cardiovascular morbidity and impaired autonomic functioning [6,7]. Altered autonomic regulation is often found in persons with depression [8]. Late life depression has also been associated with cerebral hypoperfusion [9,10] and an increased burden of cerebral white matter diseases, which can be caused by ischemic damages because of cerebral hypoperfusion [11]. Aging-related blood vessel changes include narrowing, being calcified and tortuous, and thereby a reduction in cerebral perfusion [6]. A four-year longitudinal study revealed that approximately 10% of older adults with symptomatic OH develop depression [6]. Therefore, OH significantly impairs health-related quality of life (HRQoL) [12,13]. However, there remains limited evidence on the effect of OH management programs on psychological health outcomes.

Both pharmacological (e.g., midodrine) and non-pharmacological interventions (e.g., water intake, physical exercise, or head elevation) have been used to treat OH [14]. However, these approaches mainly aim to relieve OH-related symptoms temporarily, rather than addressing ANS imbalance, which is the underlying mechanism of OH among older adults. Heart rate variability (HRV) biofeedback is a non-pharmacological intervention used to modulate autonomic cardiac regulation by increasing HRV, reinstating control over cardiac vagal function, and thus improving the perception of self-regulation [15].

HRV biofeedback training, which was a core component of the BBIP, is a breathing-based cardiorespiratory feedback training that decreases respiratory rates. This breathing pattern stimulates the baroreceptor and facilitates autonomic balance by the baroreflex gain [16]. Breathing in the resonance frequency (resonance between respiratory and baroreflex rhythms) and slow effortless breathing training stimulates the reflexes of the cardiovascular system; in particular, the baroreflex enhances autonomic balance in the ANS [17,18]. Given that autonomic balance is defined as complex heart rate patterns that change in response to respiratory fluctuations, the body can be brought into the state of autonomic balance through guided breathing with HRV biofeedback [19].

HRV biofeedback has proven to be effective in managing cardiovascular diseases, including hypertension and coronary artery diseases, as well as other chronic conditions [10]. More recently, Park et al. reported that a biofeedback-based multicomponent program including lifestyle modification successfully improved the ANS balance and the urinary symptoms and sleep patterns of older adults with overactive bladder syndrome. Group education about modifying lifestyle as well as telephone counseling were provided to enhance the effects of biofeedback, as reports have found that these approaches influence OH and/or ANS function [20].

Therefore, we developed a biofeedback-based integrated program (BBIP), a multicomponent intervention focusing on using HRV biofeedback to improve OH in community-living older adults. Previously, BBIP was shown to successfully improve OH and associated symptoms by modulating the ANS [21].

The purpose of this secondary analysis study is to identify the impact of BBIP on improving psychological health outcomes, including HRQoL, fall efficacy, depression, and anxiety, among community-residing older adults with OH.

## 2. Materials and Methods

### 2.1. Study Design

This study was a non-randomized controlled pilot trial designed to examine the effectiveness of the 12-week BBIP on psychological health outcomes in older adults with OH residing in a community setting. The trial was registered in a Korean registry of the WHO International Clinical Trials Registry Platform (ICTRP) (trial registration number: ClinicalTrials.gov KCT0004060).

A non-RCT design was chosen for this study because many older adults in the community spend their time at senior welfare centers, making it challenging to contact individual volunteers for randomization. In addition, we needed to recruit volunteers willing to participate in group activities. Maintaining uniform environmental conditions such as room temperature with a minimum level of noise and light was crucial for producing reliable findings and maximizing the effect of HRV biofeedback. For these reasons, we contacted senior welfare centers instead of individual older adults, which made random assignment of participants difficult.

### 2.2. Participants and Setting

The inclusion and exclusion criteria for participants, as well as the recruitment procedure, are detailed in a prior publication [21]. Briefly, participants were eligible if they (1) were 65 years or older, (2) met the diagnostic criteria for OH, (3) were able to perform activities of daily living independently, (4) had Mini-Mental State Examination scores of 24 or higher, and (5) had not participated in exercises or therapeutic programs aimed at relieving OH symptoms within the past three months. The diagnostic criteria for OH were defined as follows: for those without hypertension, a decrease in systolic blood pressure (SBP) ≥ 20 mm Hg or diastolic BP (DBP) ≥ 10 mm Hg; for those with hypertension, a decrease in SBP ≥ 30 mm Hg or DBP ≥ 15 mm Hg, within three minutes of standing upright after measuring the mean BP for five minutes while in a lying position [22]. Exclusion criteria included participants with dizziness related to current illnesses, a history of neuropsychiatric diseases that could disrupt autonomic function, diagnosed psychological illnesses or autonomic neuropathy, and those with a history of medication that could cause OH, ensuring the study focused exclusively on age-related OH.

### 2.3. Sample Size Estimation

A power analysis using G*power version 3.1.9, with two groups, a significance level of 0.05, power of 0.80, and an effect size (d) of 0.86 [23], required 23 participants per group. We recruited 30 participants per group to account for a potential dropout rate of 20%.

### 2.4. Recruitment and Procedures

The recruitment and sample selection process is outlined in a previous publication [11]. In brief, we contacted eight senior welfare centers, and two agreed to participate. The coin toss method was used to randomly assign these centers to the intervention and control groups. To recruit participants, we utilized banners, flyers, and informational meetings. Before the initiation of the BBIP, baseline tests were conducted, including BP measurement and self-reports of HRQoL, fall efficacy, depression, and anxiety. For participants unable to read the questionnaires, study team members verbally administered the questions and recorded their responses.

### 2.5. Intervention

The 12-week BBIP was developed based on a comprehensive literature review regarding OH management for older adults [24,25]. An expert group consisting of five clinical and research specialists with expertise in OH and geriatric care assessed the content validity. The intervention was revised and finalized based on the expert group’s recommendations.

Details about the BBIP are presented in a previous publication [21]. The intervention consisted of three components: (1) HRV biofeedback training, which was the core component aimed at balancing the ANS; (2) a weekly group education session providing knowledge on OH management, including lifestyle modifications related to diet, medication management, and physical activities to alleviate OH symptoms; and (3) telephone counseling. The intervention group met once a week at the intervention center for a 50 min group education session and individualized HRV biofeedback training. Two trained registered nurses (RNs) conducted the HRV biofeedback training using a computer biofeedback system (Procomp, Thought Technology, Montreal, QC, Canada). Participants regulated their respiration rates by monitoring visual feedback on their heart rate rhythm displayed on the screen. At baseline, the research team specified each participant’s target respiration rate, allowing them to breathe effortlessly within a range of 4 to 7 breaths per minute while the biofeedback system maintained a low-frequency-to-high-frequency ratio of approximately 1.5, which is close to an optimal sympathovagal balance. If a participant reported dizziness or difficulty breathing, the target respiration rate was modified. As the program progressed, participants became more comfortable breathing at their target rate.

Additionally, daily logs were reviewed in which participants recorded their lifestyle modification practices based on individualized goals set during the group education session. As positive reinforcement, a small monetary reward was granted to the participant who most successfully achieved their individualized goals in the 6th week of the intervention. Telephone counseling was provided for 10 min, twice a week, on weekdays to increase compliance and address problems or questions related to OH management. Each participant also received a pedometer and an elastic exercise band to encourage physical activity.

After the baseline tests, the control group received one 30 min education session on managing OH in daily life at the beginning of the study period. An elastic exercise band and a pedometer were provided to encourage physical activity. Follow-up tests were conducted after 12 weeks. The Institutional Review Board (IRB) of K University approved this study (approval number: 40525-201809-BR-87-03). Participants were provided with both verbal and written explanations of the study’s purpose and procedures, assured of voluntary participation, and informed that withdrawal would not result in any disadvantage. It was also explained that collected data would be analyzed anonymously and would be destroyed three years after the study’s completion.

### 2.6. Outcome Measurements

#### 2.6.1. OH

Beat-to-beat BP was measured at baseline and 12 weeks using a beat-to-beat digital photo plethysmography system, the Finometer Pro (Finapres Medical Systems BV, Amsterdam, The Netherlands). Upon arrival, participants rested for 5 min in a sitting position, after which an RN took their BP in a supine position for 5 min. They then stood up, and BP was monitored for 3 min. BP signals were recorded and analyzed using BeatScope Beat Analysis softwasre for Windows NT/9x/2000 ( (https://www.finapres.com, accessed on 26 June 2019) Finapres Medical Systems BV, The Netherlands).

#### 2.6.2. HRQoL

HRQoL was measured using the EuroQol-5D-3-dimensional version scale (EQ-5D-3L) [26] (The EuroQol group, 1990). This instrument consists of 5 dimensions (mobility, self-management, daily activities, pain/discomfort, and anxiety/depression) that assess subjective current health status. Each dimension of the EQ-5D-3L allows participants to choose from “No problems,” “Some problems,” and “Severe problems.” Participants’ individual perception of their general health status was also scored using the EQ-VAS, a visual analog scale ranging from 0 to 100, where higher scores indicate better perceived health. This scale has been validated and is reliable for measuring both general and disease-specific HRQoL [27]. Quality weight tariffs specific to Korean populations were used to score the EQ-5D-3L [28]. Additionally, the minimal clinically important difference (MCID) of the EQ-5D-3L was calculated using an anchor-based change difference approach for each group. The MCID represents the smallest change in a patient-reported outcome measure that participants perceive as clinically meaningful over time or following an intervention [29].

#### 2.6.3. Depression

Depression was assessed using the Korean version of the Geriatric Depression Scale (GDS)-Short Form [30,31]. This instrument comprises 15 items that assess mood over the past week with yes/no responses. The Cronbach’s alpha coefficient was 0.88 [31].

#### 2.6.4. Anxiety

A Korean version of the State–Trait Anxiety Inventory (STAI) [32,33] was used to measure the level of anxiety in participants. The 20-item STAI includes both state and trait components, quantifying anxiety symptoms related to diseases. The STAI-state subscale assesses temporary anxiety symptoms due to specific situations, whereas the STAI-trait subscale examines inherent tendencies to experience anxiety symptoms. Each item has four response options generating a total score (range: 20–80), with higher scores indicating greater levels of anxiety symptoms.

#### 2.6.5. Fall Efficacy

The 12-item Korean version of the Fall Efficacy Scale-International, originally developed by Yardley et al. [34] and translated and validated by Huh et al. [35], was used to assess fall efficacy. Participants self-reported their confidence in performing daily activities without falling, using a 4-point Likert scale (1 = not confident, 4 = very confident). The Cronbach’s alpha was 0.96 in Huh et al.‘s study [35] and 0.91 in this study.

### 2.7. Statistical Analysis

Data were entered and analyzed using IBM SPSS version 26.0 (IBM SPSS Statistics for Windows, Version 26.0. IBM Corp, Armonk, NY, USA). The Shapiro–Wilk test was conducted to test the normal distribution of the sample. Because the test results suggest a non-normal distribution of the sample, non-parametric tests including Fisher’s exact tests, Wilcoxon signed rank tests, and Mann–Whitney U tests were carried out to examine homogeneity, and the effectiveness of BBIP. McNemar tests were performed to compare percentage changes in participants reporting some or severe problems across the dimensions of EQ-5D. *p* values < 0.05 were considered statistically significant. Regarding the MCID estimation of EQ-5D-3L, change scores from 12 weeks to the baseline of 7 to 17 points on the EQ-VAS were used as an anchor-based range for minimal improvements, as suggested by Jehu et al.’s study involving older adults with a history of falls [36]. The anchor-based range for maximal improvements was defined as ≥18 points, −6 to 6 points for no meaningful difference, −7 to −17 for minimal declines, and ≤−18 points for maximal declines.

Sensitivity to the results was evaluated by applying three methods for handling missing values: mean imputation, zero imputation, and removal of missing values. The analyses included all major variables containing missing values (GDS, STAI, fall efficacy, EQ-VAS, and EQ-5D) as well as the change values between the baseline and 12 weeks.

## 3. Results

### 3.1. Baseline Characteristics of Participants

**Figure 1:**
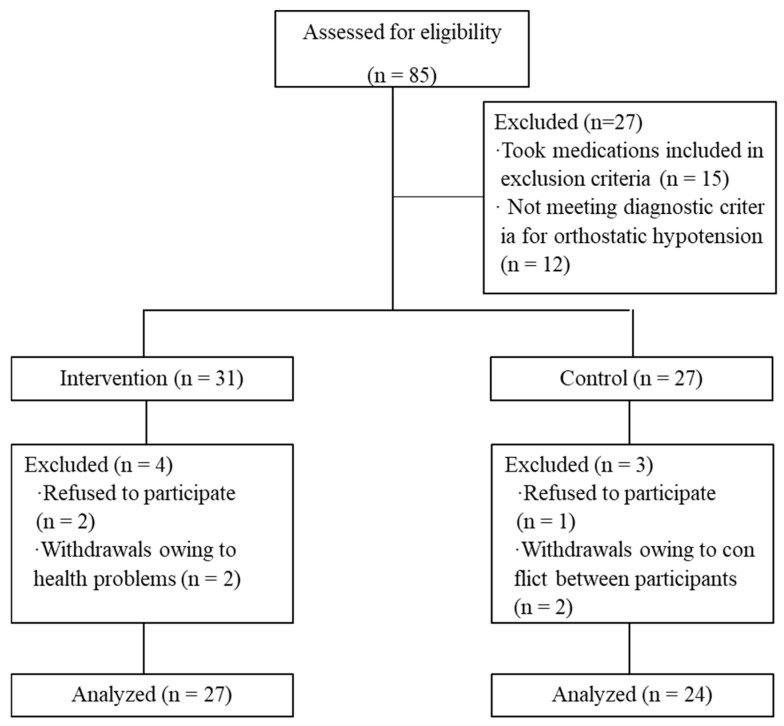
Selection process for study participants. From Han et al. (2023) [21].

Eighty-five individuals were initially enrolled and participated in screening tests (45 in the intervention group and 40 in the control group). After excluding 14 participants from the intervention group and 13 from the control group who did not meet the inclusion criteria, 31 and 27 participants remained in each group, respectively. Before the completion of the 12-week BBIP, there were seven attritions unrelated to the study (four in the intervention group and three in the control group). Finally, data from 27 participants in the intervention group and 24 participants in the control group were included in the analysis (Figure 1).

Details regarding the demographic characteristics of participants were described previously [21]. Participants in the intervention group had an average age of 82.04 years (SD = 4.48), while those in the control group had an average age of 79.38 years (SD = 5.09), with no significant difference between groups (*p* = 0.87). All participants were women, and more than half had hypertension as a comorbid condition. At baseline, the change in systolic BP after postural changes was 37.44 mmHg (SD = 13.14) in the intervention group and 37.50 mmHg (SD = 16.26) in the control group (*p* = 0.109). The differences in diastolic BP after postural changes were 17.59 mmHg (SD = 12.88) in the intervention group and 13.96 mmHg (SD = 11.38) in the control group, respectively, at baseline (*p* = 0.111). There were no significant differences in other demographic and health-related characteristics between the groups at baseline (Table 1).

As a result of the 12-week BBIP administration, the drop in both systolic BP and diastolic BP after postural changes significantly decreased in the intervention group. The HRV analysis demonstrated that the intervention group also showed improvement in ANS activity, and orthostatic symptom scores significantly decreased. In contrast, the control group did not exhibit any significant improvement in the outcome variables [21].

### 3.2. Changes in HRQoL, Depression, Anxiety, and Fall Efficacy

**Figure 2:**
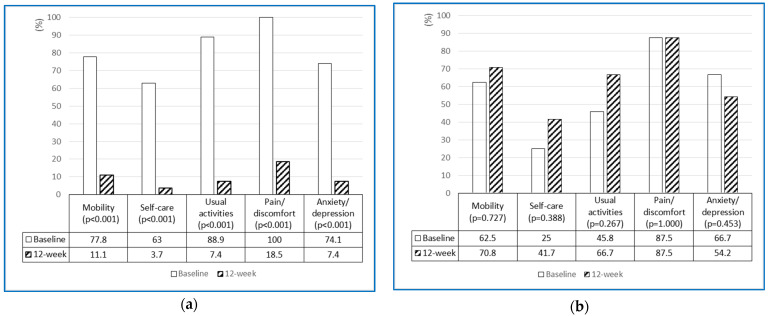
(**a**) Percentage changes in the intervention group reporting some or severe problems across the dimensions of EQ-5D-3L (n = 27). (**b**) Percentage changes in the control group reporting some or severe problems across the dimensions of EQ-5D-3L (n = 24).

Table 2 presents the changes in psychological outcomes between the baseline and 12 weeks after the administration of the BBIP. The level of HRQoL significantly improved in the intervention group (*p* < 0.001), whereas the control group experienced a slight reduction in HRQoL (*p* = 0.188). When analyzed by dimensions of the EQ-5D-3L, the percentages of participants reporting some or severe problems significantly decreased in the intervention group across all five dimensions, while the control group showed no significant changes (Figure 2a,b). However, both the intervention and control groups significantly increased EQ-VAS scores at 12 weeks (*p* < 0.001 and *p* = 0.016, respectively).

Table 3 presents MCID estimates using an EQ-VAS as an anchor. In the BBIP group, approximately 81.5% (n = 22) reported above the minimal improvement in EQ-5D whereas 66.6% (n = 16) showed minimal or maximal improvement.

The intervention group also demonstrated a significant decrease in depression (*p* < 0.001) and anxiety (*p* < 0.001). In contrast, the control group showed a slight improvement in depression and an increase in anxiety, but neither change was statistically significant (*p* = 0.215 and *p* = 0.133, respectively). Additionally, findings related to fall efficacy revealed a significant increase in the intervention group (*p* < 0.001), while no significant changes were observed in the control group (*p* = 0.511) (Table 2).

The effect sizes of variables were found to be small to moderate (EQ-5D = 0.68; EQ-VAS = 0.44; GDS = 0.51; STAI = 0.45; fall efficacy = 0.64). The sensitivity analysis results showed statistically significant differences between the main variables across all missing value handling methods.

## 4. Discussion

This study found that 12 weeks of BBIP administration significantly improved psychological health outcomes of older adults with OH who reside in a community setting. In the initial study, we presented the direct effect of BBIP on relieving OH and improving autonomic nervous system function. This secondary data analysis demonstrated that BBIP effectively improved psychological health outcomes, including health-related quality of life (HRQoL), depression, anxiety, and fall efficacy among participants. The analyses of both the original study and the substudy provide a new understanding that alleviating OH through BBIP administration may lead to improvements in the psychological health of affected older adults. This study is significant because it allows us to make full use of existing data to explore questions not addressed in the original study and to broaden the research scope [37].

Overall HRQoL was significantly increased in the intervention group. Notably, the analysis of EQ-5D dimensions revealed that participants in the experimental group significantly decreased their reports of some or severe problems in all five domains of the EQ-5D (Figure 2a), whereas the control group tended to have either maintained or increased problems (Figure 2b). Although there is limited literature reporting on HRQoL for individuals with OH, Kim and colleagues [13] found that older adults with OH experienced significantly greater problems in HRQoL compared to those without.

The baseline characteristics of the EQ-5D were similar among participants in this study and those in Kim et al.’s study [13], as a majority of participants reported some or severe problems in the dimensions of mobility, usual activities, pain/discomfort, and anxiety/depression. The finding that the intervention group had significantly decreased problems across all five dimensions suggests that improving OH through the BBIP alleviated symptoms related to OH and consequently increased HRQoL. Interestingly, while the control group showed a slight decrease in EQ-5D scores at 12 weeks (though not significant, *p* = 0.188), the EQ-VAS score significantly increased from 53.13 ± 17.05 to 61.67 ± 16.59 (*p* = 0.016). This discrepancy may be explained by the conceptual differences between EQ-5D and EQ-VAS. The EQ-5D is designed to assess HRQoL by providing a profile of an individual’s functioning, whereas the EQ-VAS score reflects an overall rating of health from the respondent’s perspective [38]. Furthermore, the control group’s participation in certain health promotion programs at the study site, coupled with the lack of blinding to group assignments, may have contributed to an improved perception of subjective health status at 12 weeks. Further experimental studies are needed to compare the changes in EQ-5D and EQ-VAS scores.

Anchor-based MCID estimates for the EQ-5D-3L suggest that participants may perceive a clinically meaningful improvement with a change score of at least 0.289 following BBIP. Although Revicki et al. suggested that the MCID corresponds to the β-coefficients generated from a linear regression model (ordinary least squares), previous studies have demonstrated convergence among anchor-based MCID change differences, anchor-based regression, and distribution-based approaches. The MCID change difference in this study is much higher than those found in Jehu et al.’s study [36] involving older adults with a history of falls, which used VAS as an anchor. A critical review reported that estimates of the MCID range from 0.03 for patients with low back pain [39] to 0.52 for patients with lumbar stenosis [40], and the findings of this study fall within this range. However, the interpretation of MCID estimates requires the consideration of population characteristics, treatment type, and follow-up periods [41]. Further studies need to be conducted to investigate clinically meaningful differences in EQ-5D scores among older adults with orthostatic hypotension (OH) following BBIP.

Regarding other psychological health outcomes, the intervention group exhibited significant improvements in symptoms of depression and anxiety, as well as in fall efficacy, while the control group did not show any significant changes. Fall efficacy measures the fear of falling, as this fear is referred to as “low perceived self-efficacy in avoiding falls during essential, non-hazardous activities of daily living” [42]. According to Arik and colleagues [43], individuals with OH reported moderate to high levels of fear of falling, and a higher level of fear of falling has been associated with decreased QoL [44,45], greater depression, inactivity, and functional decline [46]. Thus, these findings suggest that the BBIP could contribute to improving physical and psychological well-being by enhancing fall efficacy.

The close relationship between OH and depression is supported by previous studies [13,47]. Briggs and colleagues [10] reported that older adults with symptomatic OH have a two-fold higher risk of developing depression in a longitudinal study with a four-year follow-up period. Later-life depression has been linked to a loss of integrity in the frontal lobe, which is sensitive to hypoperfusion [48,49], as well as to altered autonomic regulation, as demonstrated by lowered HRV [6]. Long-standing psychological distresses, such as anxiety and depression, can induce physiological stress and subsequently disturb stability of the ANS—lowering vagal control and hyperarousal--allowing overactivation in sympathetic tone [19].

Therefore, the decrease in depression observed in the intervention group may be attributed to improved OH-related cerebral hypoperfusion and ANS. Additionally, the social interaction fostered during weekly group education sessions and telephone counseling may have also played a role in alleviating depression.

Baseline STAI scores indicated that participants experienced a high level of anxiety; however, after 12 weeks, the intervention group significantly lowered their anxiety levels, while the control group did not show any significant change. Some literature suggests a positive correlation between OH and anxiety disorders, as evidenced by poorer SBP or DBP responses to orthostasis in individuals with trait anxiety [50] or anxiety disorders [51]. Other studies have reported the therapeutic effects of biofeedback on psychological health issues, including anxiety and depression [52,53]. Consequently, the reduced anxiety levels may be attributable to both the effects of biofeedback and the improvements in OH resulting from the BBIP administration.

Limitations of this study should be noted. First, participants had to be capable of performing activities of daily living independently and able to visit study sites. Consequently, there is limited information regarding the effectiveness of the intervention in older adults with physical or cognitive impairments. Second, using a convenience sampling-based non-RCT strategy may have introduced selection bias. Although we did not exclude men, all participants were women, possibly because men might be reluctant to participate in group activities, which were a major part of BBIP, given that women are predominant in senior centers in South Korea. However, randomization does not always reduce the influence of confounding variables, especially when the sample size is small [54]. Given the characteristics of the participants, who were community-residing Asian older adults, and the nature of the intervention, such as group exercise and education, the high compliance and low attrition rates in this study might be attributed to the use of a non-RCT design.

The small sample size and lack of blinding may threaten the internal validity of the study findings, particularly since the study measures psychological health outcomes that depend on the subjective evaluation of participants. Awareness of group assignment could influence participants’ responses. A study with a larger sample size and blinding may yield different findings. Despite these limitations, to our knowledge, this study is the first trial demonstrating the promising effects of HRV biofeedback combined with group education and individual counseling on improving psychological health, including HRQoL, anxiety, depression, and fall efficacy, in community-dwelling older adults with OH.

## 5. Conclusions

This study found that the BBIP significantly improved HRQoL, depression, anxiety, and fall efficacy in older adults with OH by providing a multicomponent intervention focused on HRV biofeedback. Future studies should evaluate the long-term implications of the BBIP by examining psychological effects using an RCT design and a larger sample size to minimize selection bias.

## Figures and Tables

**Table 1:** Homogeneity test results at baseline (N = 51).

Variables	Category	Intervention (n = 27) n (%) or M ^1^ ± SD ^2^	Control (n = 24) n (%) or M ± SD	*p*
Age (years)		82.04 ± 4.48	79.38 ± 5.09	0.087
Education	None	11 (40.7)	10 (41.7)	0.946
	≥Elementary	16 (59.3)	14 (58.3)	
Smoking	Yes	2 (7.4)	1 (4.2)	0.545
	No	25 (92.6)	23 (95.8)	
Number of medications	0–1	15 (55.6)	10 (41.7)	0.351
	≥2	12 (44.4)	14 (58.3)	
Comorbid conditions (multiple responses)	Hypertension	18 (66.7)	20 (83.3)	0.695
	Diabetes	5 (18.5)	7 (29.2)	
	Others	10 (37.0)	13 (54.2)	
Experience of falls	Yes	2 (7.4)	3 (12.5)	0.656
	No	25 (92.6)	21 (87.5)	
Changes in BP ^3^ with postural change (mmHg)	△SBP ^4^	37.44 ± 13.14	37.50 ± 16.26	0.109
	△DBP ^5^	17.59 ± 12.88	13.96 ± 11.38	0.111

Footnote. ^1^ M = Mean; ^2^ SD = Standard deviation; ^3^ BP = Blood pressure; ^4^ ΔSBP = Change in systolic blood pressure; ^5^ ΔDBP = Change in diastolic blood pressure.

**Table 2:** Changes in psychological health outcomes after 12 weeks of BBIP administration (N = 51).

Variables	Intervention (n = 27)			Control (n = 24)		
	Baseline M ± SD	12 Weeks M ± SD	*p*	Baseline M ± SD	12 Weeks M ± SD	*p*
EQ-5D ^1^	0.39 ± 0.28	0.83 ± 0.18	<0.001	0.48 ± 0.30	0.39 ± 0.28	0.188
EQ-VAS ^2^	51.67 ± 21.79	77.93 ± 16.08	<0.001	53.13 ± 17.01	61.67 ± 16.60	0.016
GDS ^3^	8.33 ± 2.53	2.37 ± 2.31	<0.001	7.83 ± 3.44	7.13 ± 5.03	0.215
STAI ^4^	48.96 ± 5.67	37.26 ± 7.54	<0.001	45.83 ± 10.64	50.42 ± 16.81	0.133
Fall efficacy	29.78 ± 2.29	37.37 ± 5.24	<0.001	30.62 ± 2.14	31.25 ± 4.71	0.511

Footnote. ^1^ EQ-5D = EuroQoL-5 Dimensions; ^2^ EQ-VAS = EuroQoL Visual Analogue Scale; ^3^ GDS = Geriatric Depression Scale; ^4^ STAI = State–Trait Anxiety Inventory.

**Table 3:** Anchor-based MCID improvements and declines on the EQ-5D-3L.

Group	Change	Anchor-Based Change Difference MCID (SD)	MCID Range
BBIP (n = 27)	Max improvement (n = 18)	0.447 (0.299)	−0.006~0.752
	Min improvement (n = 4)	0.289 (0.191)	0.037~0.498
	No meaningful change (n = 3)	0.429 (0.336)	0.080~0.752
	Max decline (n = 1)	0.663 (−)	-
	Max decline (n = 1)	0.752 (−)	-
Control (n = 24)	Max improvement (n = 8)	−0.058 (0.398)	−0.663~0.583
	Min improvement (n = 8)	−0.122 (0.350)	−0.672~0.418
	No meaningful change (n = 4)	−0.158 (0.110)	−0.254~0.000
	Max decline (n = 2)	0.440 (0.263)	0.254~0.626
	Max decline (n = 2)	−0.0089 (0.364)	−0.672~−0.288

## Data Availability

The data presented in this study are available on request from the corresponding author due to privacy of participants.
